# Plasmacytoid urothelial carcinoma of renal pelvis with positive zinc finger E–box–binding homeobox 1: a case report

**DOI:** 10.1186/s13000-020-01043-6

**Published:** 2020-10-08

**Authors:** Atsuko Takada-Owada, Yumi Nozawa, Masato Onozaki, Shuhei Noda, Tsengelmaa Jamiyan, Yuumi Tokura, Yoshimasa Nakazato, Takao Kamai, Kazuyuki Ishida

**Affiliations:** 1 Department of Diagnostic Pathology, Dokkyo Medical University, 880 Kitakobayashi, Mibu, Tochigi 321-0293 Japan; 2 Department of Urology, Dokkyo Medical University, Mibu, Tochigi Japan

**Keywords:** Plasmacytoid urothelial carcinoma, CD38, CD138, Epithelial–mesenchymal transition, Zinc finger e–box–binding homeobox 1

## Abstract

**Background:**

The tumor transformation mechanism of a plasmacytoid urothelial carcinoma remains unexplained. We describe the case of a plasmacytoid urothelial carcinoma of the renal pelvis in which the expression of zinc finger E–box–binding homeobox 1 (ZEB1), a key nuclear transcription factor in an epithelial–mesenchymal transition, is involved in tumor transformation.

**Case presentation:**

The patient had a left nephrectomy with the clinical diagnosis of left pelvic renal cancer. The resected specimen showed that the tumor surface comprised a noninvasive papillary urothelial carcinoma with the carcinoma in situ, and the invasive area comprised a plasmacytoid urothelial carcinoma characterized by the presence of single dyscohesive malignant cells that resembled plasma cells in a loose myxoid stroma. The noninvasive urothelial carcinoma was positive for cytokeratin and E–cadherin, and negative for vimentin and ZEB1. In contrast, the invasive plasmacytoid urothelial carcinoma was positive for cytokeratin and also vimentin and ZEB1, and negative for E–cadherin. Additionally, this component was immunoreactive for CD138 and CD38 that are immunohistochemical markers for plasma cells.

**Conclusion:**

We suggest that ZEB1 is involved in the plasmacytoid transformation by repressing the E–cadherin in a plasmacytoid urothelial carcinoma.

## Background

Plasmacytoid urothelial carcinoma, which is a characteristic morphology similar to plasma cells, is an unusual variant that is included in the World Health Organization classification of urothelial neoplasms [1, 2]. Several studies have recently reported that the plasmacytoid component of a urothelial carcinoma exhibits positive staining for CD138, an immunohistochemical marker for plasma cells, in addition to showing a morphological similarity [3–6]. A plasmacytoid urothelial carcinoma is an aggressive subtype associated with a poor prognosis even if the plasmacytoid component is locally limited [7]. However, the reason for plasmacytoid differentiation in a urothelial carcinoma being associated with a poor prognosis has yet to be explained.

The epithelial–mesenchymal transition (EMT) is related to the progression of a carcinoma towards dedifferentiation and more malignant states [8]. A recent study has identified a specific molecular mechanism underlying EMT [9]. In particular, zinc finger E–box binding homeobox 1 (ZEB1) is a key nuclear factor that specifically binds to and represses the promotor region of E–cadherin, suggesting that a decreased expression of E–cadherin could occur in tumor cells [9, 10]. In addition, immunohistochemically, the expression of ZEB1 reduced E–cadherin expression in the sarcomatous component of other cancers [11]. In contrast, although it has been reported that a plasmacytoid urothelial carcinoma loses E–cadherin expression [12], there have been no reports stating whether or not ZEB1 contributes to the tumorigenesis of a plasmacytoid urothelial carcinoma.

Here we report a patient with a plasmacytoid urothelial carcinoma of the renal pelvis whose plasmacytoid component was ZEB1 positive as determined by immunohistochemistry.

## Case presentation

### Clinical history

A 73-year-old man, who had previously presented with angina and interstitial pneumonia, developed left lower back pain and attended our hospital. Peri–pelvic extravasation of urine was observed with magnetic resonance imaging. Contrast–enhanced computed tomography confirmed a left renal pelvic tumor. The patient’s tumor marker serum levels, such as those for carcinoembryonic antigen, carbohydrate antigen 19–9, and squamous cell carcinoma, were within the normal range. No malignant cells were identified in the patient’s urine cytology. A left nephroureterectomy was subsequently performed with a clinical diagnosis of left pelvic renal cancer.

Left nephroureterectomy specimens were obtained that were originally prepared from 10% buffered formalin–fixed, paraffin–embedded tissue according to our routine hospital procedure. A histopathological examination was performed using hematoxylin and eosin staining. Immunohistochemistry was conducted using an autoimmunostainer (Leica BOND–III system: Leica Biosystems, Newcastle, UK). The antibodies we employed are listed in Table 1.

**Table 1:** Antibodies used for immunohistochemical study

Antibody	Clone	Dilution	Source
Cytokeratin AE1/AE3	AE1 and AE3	Ready to use	Leica biosystems, Newcastle, UK
E-cadherin	36B5	Ready to use	Leica biosystems, Newcastle, UK
Vimentin	V9	Ready to use	Leica biosystems, Newcastle, UK
CD138	SPC32	1:200	Leica biosystems, Newcastle, UK
CD38	MI15	Ready to use	Leica biosystems, Newcastle, UK
ZEB1	polyclonal	1:100	Sigma-Aldrich, St. louis, MO

### Pathologic findings

**Fig. 1:**
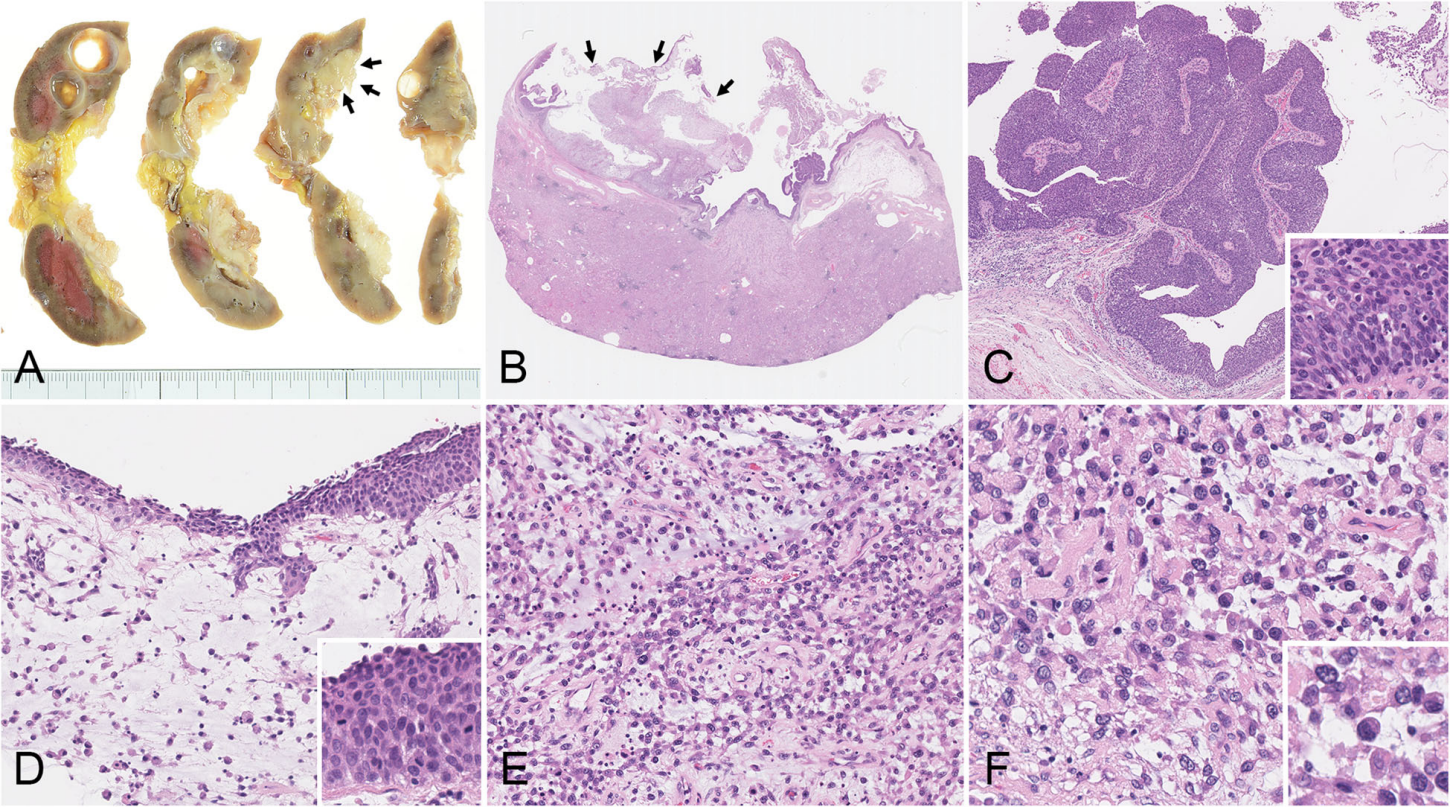
Macroscopic and histological findings for a plasmacytoid urothelial carcinoma. **a** Gross examination of the cut surface of a nephrectomy specimen shows a whitish solid tumor with a partially myxoid change in the renal pelvis (arrows). **b** The tumor was mainly located in the renal pelvic mucosa and exhibited two distinct morphological components. **c** The tumor surface comprised a noninvasive urothelial carcinoma, including a high–grade papillary urothelial carcinoma and a carcinoma in situ. **d** Transitional findings between the urothelial carcinoma in situ and the invasive plasmacytoid urothelial carcinoma were recognized. **e** In the invasive area, the plasmacytoid tumor cells in the loose myxoid stroma were dyscohesive and lacked cell adhesions. **f** The plasmacytoid tumor cells had an eccentrically placed nucleus and an abundant amphophilic to eosinophilic cytoplasm. **b**-**f**. Hematoxylin and eosin–stained sections. Original magnification: **b**, Scanning view; **c**, × 40; **d** and **e**, × 200; **f**, × 400

There was a well–circumscribed exophytic lesion in the renal pelvis that measured 42 × 25 mm. The cut surface of the tumor showed a whitish mass with a partially myxoid change (Fig. 1a). Microscope observations revealed that the tumor was mainly located in the renal pelvic mucosa (Fig. 1b). The tumor exhibited two distinct morphological components. The tumor surface comprised a noninvasive urothelial carcinoma, which included a high-grade papillary urothelial carcinoma and a carcinoma in situ (CIS) (Fig. 1c and d). Whereas the invasive urothelial carcinoma was composed of cells that were dyscohesive, lacked cell adhesion and were set in a loose myxoid stroma (Fig. 1d and e). The transition of CIS and the invasive urothelial carcinoma was seen (Fig. 1d). The invasive tumor cells had an eccentrically placed nucleus and abundant amphophilic to eosinophilic cytoplasm and exhibited a striking morphologic overlap with plasma cells (Fig. 1f). A plasmacytoid urothelial carcinoma was diagnosed based on the morphology findings.

**Fig. 2:**
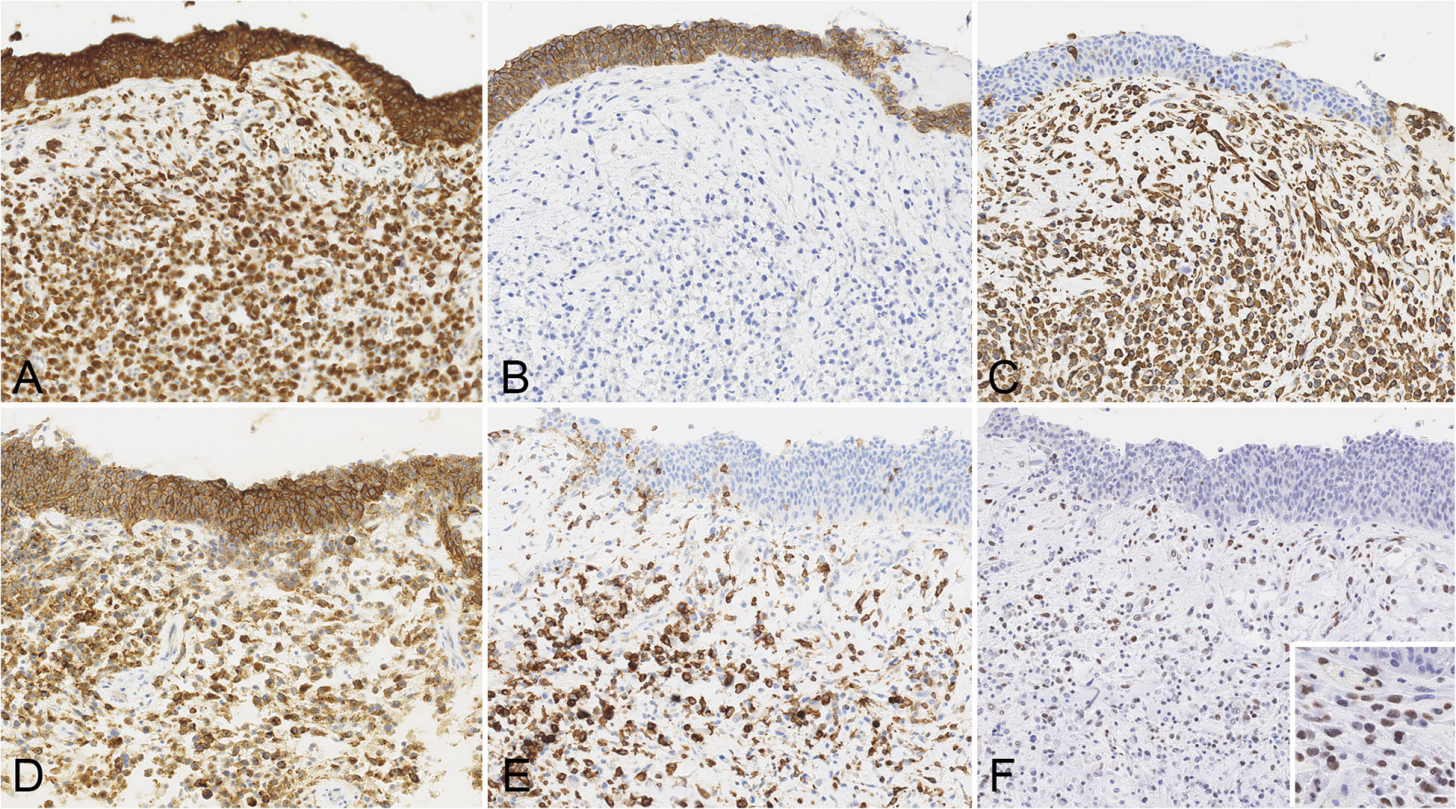
Immunohistochemical analysis of the plasmacytoid urothelial carcinoma. Immunoreactivities of cytokeratin (**a**) and E–cadherin (**b**) in the urothelial carcinoma in situ were seen. In contrast, the expression of vimentin (**c**), CD38 (**e**), and ZEB–1 (**f**) was only observed for the invasive plasmacytoid component. ZEB–1 (**f**) was expressed in the nucleus of the plasmacytoid tumor cells. CD138 (**d**) was positive for both the urothelial carcinoma in situ and the invasive plasmacytoid component. **a**-**f**. Original magnification, × 200

Immunohistochemically, the noninvasive urothelial carcinoma was positive for cytokeratin and E–cadherin, whereas it was negative for vimentin (Fig. 2a-c). CD138 and CD38, which are immunohistochemical markers for plasma cells, showed opposite immunostaining, and this noninvasive urothelial carcinoma was positive for CD138 and negative for CD38 (Fig. 2d and e). These cells were also negative for ZEB1 (Fig. 2f). On the other hand, the component of the plasmacytoid urothelial carcinoma was immunoreactive for cytokeratin, suggesting that these findings indicated the characteristics of an epithelium (Fig. 2a). However, E–cadherin was negative, and vimentin, CD138 and CD38 were positive for this component (Fig. 2b-e). In addition, ZEB1 expression was diffuse positive for this component (Fig. 2f). In summary, as shown in Table 2, the immunohistochemical determination of an invasive plasmacytoid urothelial carcinoma was characterized by E-cadherin negative, CD38 positive, and ZEB1 positive cells, unlike a noninvasive urothelial carcinoma.

**Table 2:** Immunoreactivities for two distinct morphological components

	Noninvasive urothelial carcinoma	Invasive plasmacytoid urothelial carcinoma
Cytokeratin	+	+
E-cadherin	+	-
Vimentin	-	+
CD138	+	+
CD38	-	+
ZEB1	-	+

+ , diffuse positive; -, negative

## Discussion and Conclusions

Our work revealed the following two important suggestions. Increased ZEB1 expression and the loss of E–cadherin expression are associated with the tumorigenesis of a plasmacytoid urothelial carcinoma. CD38 as well as CD138 is a useful immunohistochemical marker for the diagnosis of a plasmacytoid urothelial carcinoma.

First, ZEB1 expression and the loss of E–cadherin expression in an invasive urothelial carcinoma contributed to the plasmacytoid transformation. The mechanism of the plasmacytoid transformation in an invasive urothelial carcinoma is not well documented in the literature. Recently, it has been reported that ZEB1 is a key EMT nuclear transcription factor that plays an important role in regulating E–cadherin expression [9, 13]. In this case, the plasmacytoid tumor cells were negative for E–cadherin and positive for vimentin and ZEB1, suggesting that ZEB1 might be involved and play an important role in the tumor transformation of a plasmacytoid urothelial carcinoma through the EMT mechanism. In addition, a plasmacytoid urothelial carcinoma has a worse prognosis than a usual infiltrating urothelial carcinoma [7]. The expression of ZEB1 has been reported in various human cancers and increases the resistance of cancer cells to chemotherapy and radiation therapy [14], indicating that ZEB1 is a transcription factor not only involved in the tumorigenesis of cancers but also in the prognosis for cancer patients. The results of this case suggest that ZEB1 expression in plasmacytoid tumor cells may be associated with a poor prognosis as regards plasmacytoid urothelial carcinomas.

Second, CD38 is an appropriate immunohistochemical marker for confirming a plasmacytoid transformation in a urothelial carcinoma. Several studies have shown that the plasmacytoid tumor cells of a urothelial carcinoma are immunoreactive for CD138 [3–6]. CD138 is a highly sensitive and specific marker for normal and neoplastic plasma cells [15]. However, CD138 expression has also been observed in the urothelial epithelium and in various urothelial carcinoma cells [16]. It has been mentioned that the staining of CD138 might make it difficult when diagnosing plasmacytoid urothelial carcinomas and the other diseases [7]. In this case, CD138 immunostaining could not distinguish between the tumor transformation of the noninvasive urothelial carcinoma and the plasmacytoid component. In contrast, CD38 was positive for plasmacytoid tumor cells and negative for noninvasive urothelial carcinoma cells. Although few studies have reported the use of CD38 to diagnose plasmacytoid urothelial carcinomas, CD38 is one of the most widely used plasma cell markers [17]. Furthermore, to the best our knowledge, CD38 immunoreactivity for carcinoma cells has not been reported. CD38 immunostaining might be useful for confirming the plasmacytoid transformation in urothelial carcinomas, although it is necessary to verify whether or not CD38 expression is found only in invasive plasmacytoid urothelial carcinomas.

In conclusion, the results of the present study suggest that ZEB1 is involved in the tumor transformation in plasmacytoid urothelial carcinomas showing CD38 expression. Further investigation is needed to substantiate this finding and determine whether the ZEB1 expression is associated with a poor prognosis for a plasmacytoid urothelial carcinoma.

## Data Availability

Unstained slides of the case can be provided if required.

## Abbreviations

EMT: Epithelial–mesenchymal transition
ZEB1: Zinc finger E–box–binding homeobox 1
